# Tools for measuring patient safety in primary care settings using the RAND/UCLA appropriateness method

**DOI:** 10.1186/1471-2296-15-110

**Published:** 2014-06-05

**Authors:** Brian G Bell, Rachel Spencer, Anthony J Avery, Stephen M Campbell

**Affiliations:** 1 Division of Primary Care, School of Community Health Sciences, University of Nottingham University Park, Medical School, Queen’s Medical Centre, Nottingham NG7 2UH, England, UK; 2 NIHR Greater Manchester Primary Care Patient Safety Translational Research Centre, University of Manchester, 7th Floor, Williamson Building, Manchester M13 9PL, England, UK; 3 Centre for Primary Care, University of Manchester, 7th Floor, Williamson Building, Manchester M13 9PL, EnglandUK

**Keywords:** Primary health care, Consensus, Patient safety, Quality indicators

## Abstract

**Background:**

The majority of patient contacts occur in general practice but general practice patient safety has been poorly described and under-researched to date compared to hospital settings. Our objective was to produce a set of patient safety tools and indicators that can be used in general practices in any healthcare setting and develop a ‘toolkit’ of feasible patient safety measures for general practices in England.

**Methods:**

A RAND/UCLA Appropriateness Method exercise was conducted with a panel of international experts in general practice patient safety. Statements were developed from an extensive systematic literature review of patient safety in general practice. We used standard RAND/UCLA Appropriateness Method rating methods to identify necessary items for assessing patient safety in general practice, framed in terms of the Structure-Process-Outcome taxonomy. Items were included in the toolkit if they received an overall panel median score of ≥7 with agreement (no more than two panel members rating the statement outside a 3-point distribution around the median).

**Results:**

Of 205 identified statements, the panel rated 101 as necessary for assessing the safety of general practices. Of these 101 statements, 73 covered structures or organisational issues, 22 addressed processes and 6 focused on outcomes.

**Conclusions:**

We developed and tested tools that can lead to interventions to improve safety outcomes in general practice. This paper reports the first attempt to systematically develop a patient safety toolkit for general practice, which has the potential to improve safety, cost effectiveness and patient experience, in any healthcare system.

## Background

The publication of the Institute of Medicine’s 2000 report ‘To Err is Human’ was a seminal moment in the discussion of patient safety. The report revealed that more people in the US were dying from medical errors than from road accidents [1]. Since then an international body of literature has been produced with the majority of it focusing on secondary care services [2]. Indeed, research on primary patient safety has lagged behind that of hospital care. However, in the developed world, most healthcare interactions occur in general practice with nearly 214 million visits to family practitioners being made annually in the US and 300 million visits being made annually in the UK. Annual spending on physician and clinical services in the US in 2011 was $541.4 billion with factors such as use and intensity of services increasing at a faster rate than in secondary care. One review of the frequency of error in general practice suggested that between 5-80 safety incidents per 100,000 consultations occur [3], in the UK this would mean that 37-600 incidents occurred each day. The potential for medical error in general practice is large, but the knowledge base is limited [2].

One reason for this situation may be that general practice is thought of as inherently low-risk, so safety is not considered a critical problem. However, serious errors leading to morbidity and mortality do occur in general practice, sometimes over long periods of time. Understanding the epidemiology of hospital errors was crucial for improving safety in hospitals and gaining public support for efforts to improve safety. There needs to be a similar focus on general practice [4]. While research has been conducted to reduce prescribing errors in general practice [5], support for interventions aimed at reducing and preventing other types of error has mainly come from small pilot studies [6]. To improve safety in primary care settings, it is imperative to know what methods, tools and indicators are currently available to measure patient safety [7].

In 2011, the American Medical Association’s (AMA) ten year report concluded that major gaps remain in our understanding of primary care patient safety with virtually no credible studies on how to improve safety [2]. To address this issue, the National Institute for Health Research School for Primary Care Research (NIHR-SPCR) in the UK commissioned a project to construct a patient safety toolkit for English general practices. In this paper we describe the development of a toolkit set of measures of general practice patient safety by using the RAND/UCLA Appropriateness Method. Such consensus techniques are a well-established approach for developing measures of quality of care in healthcare, particularly in areas where high quality evidence is contested or not available [8].

## Methods

### Identification of criteria

As described in best practice documents for the RAND/UCLA Appropriateness Method [9], we conducted an extensive literature review to identify patient safety tools, sets of indicators or individual indicators of patient safety that have been or could be used in general practice (Table 1 – key sources). Two independent reviewers (RS, SC) followed Critical Appraisal Skills Programme (CASP) and Preferred Reporting Items for Systematic Reviews and Meta- Analyses (PRISMA) guidelines [10,11] for systematic reviews. For a list of the search terms, see the Additional file 1. The following databases were searched on the first of November 2011: *Pubmed*, *Medline (Ovid 1996 onward), Embase, CINAHL, Health Management Information Consortium* and *Web of Science*. The resulting studies were too heterogeneous to conduct a meta-analysis, so the authors used a taxonomy-based approach to extract information from the identified tools, sets of indicators or individual indicators to create statements that could be rated by an expert panel.

**Table 1:** Key sources of statements for the RAND consensus panel

**Source**	**Description**	**Relevance to a toolkit for patient safety in general practice**
PMCPA Primary Medical Care Provider Accreditation [12]. Multiple statements and indicators regarding quality, some also address safety issues.	The RCGP (Royal College of General Practitioners) commissioned the University of Manchester to begin work on this quality assessment scheme in 2007. PMCPA version 1 has been piloted in 36 UK practices, half of the practices achieved ≥90% on core criteria, 9 practices achieved 100%. The data from the pilot shows that practices were able to meet the criteria.	The researchers liaised with the CQC (Care Quality Commission) to ensure that the development of PMPCA was relevant to future national aims for general practice.
EPA European Practice Assessment [13]. 57 quality statements relating to general practice.	A framework for general practice management made up of quality indicators shared by six European countries. Indicators were derived from a two-round postal Delphi questionnaire in general practice settings in Belgium, France, Germany, The Netherlands, Switzerland and the United Kingdom using the Rand Appropriateness Method.	A number of the indicators might be considered in a safety context, this work was also used to inform the PMCPA. TOPAS Europe are a Dutch organisation who are implementing and extending EPA, see; http://www.topaseurope.eu/
Multiple prescribing literature sources [14]	RAND consensus output and documents from UK organisations such as NPSA (National Patient Safety Agency) and BNF (British National Formulary). Reports of trials of interventions to improve medications management.	Results of a RAND process focusing exclusively on indicators that are drug specific will be published separately, indicators in relation to safety of prescribing systems (especially electronic systems) and medicines management were considered here.
Multiple resources on interface of general practice and secondary care e.g [15]	Focus on the literature relevant to general practice, mostly small intervention pilot studies and guidelines.	Indicators were only considered if they were under the direct control of the family practitioner, for example, offering a review post discharge.

Potential criteria that described good practice in patient safety in general practice were identified by both reviewers during data extraction. The statements were taken verbatim from the source document or derived from the key finding of the paper. Inclusion and exclusion criteria are shown in Table 2. The summary output from the literature review was condensed into a 55-page evidence booklet for panellists that described the key features of the 120 tools that were identified in the review (Panellists were sent the evidence booklet to read prior to the commencement of round 1).

**Table 2:** Inclusion and exclusion criteria

Inclusion criteria	
1.	The tools, sets of indicators or individual indicators represent good practice in the field of patient safety in primary care
2.	Preferred tools, sets of indicators or individual indicators that could be extracted electronically
Exclusion criteria	
1.	We excluded tools, sets of indicators or individual indicators that describe a pattern of care that is so unusual in UK general practice that the yield is likely to be too low to justify inclusion in the RAND process i.e. items relating to general anaesthesia
2.	Tools, sets of indicators or individual indicators seen as statutory in the UK were excluded (i.e. items relating to the introduction of an electronic health record (EHR)

From this literature, we generated a list of identified tools that were categorised according to the taxonomy of structures, processes, and outcomes [16,17]. In addition, we developed a new taxonomy (see Table 3) that classified safety tools, sets of indicators or individual indicators into dimensions of patient safety. The new taxonomy was based on previous conceptual work on quality of care [17]. Our overall focus was on the avoidance, prevention, and amelioration of adverse outcomes or injuries stemming from the processes of health care [18].

**Table 3:** A new taxonomy of patient safety in general practice

	**Safety structure & systems (Structure)**	**Patient-centred safety (Process)**	**Consequences of ‘safety’ (Outcome)**
Accessibility	Availability *(Includes; systems of access, appointment availability standards, triage, physical access)*	Availability	User Evaluation *(Includes; patient satisfaction questionnaires)*
Safety	Background Systems *(Includes; informatics, EHR, risk registers, information flow – results systems)*	Safety of clinical care *(includes; diagnosis, investigations, prescribing, treatment, follow-up: including diarised activity, referrals, discharges, interface and pathways)*	Adverse Events/Errors *(includes; mortality, incident reports, significant event (audits))*
	Management *(Includes; governance)*	Safety of Interpersonal care *(includes; communication monitoring and health literacy)*	User Evaluation *(Includes; PROMs/PREMs)*
	Premises *(Includes; equipment, devices, car parking if on site, health and safety)*		
	Learning Organisation *(Includes; knowing the needs of the practice population/community, safety culture/climate and attitudes to patient safety)*		Harm Improvement *(Includes; complaints handling, SEA outcomes, responding to error)*
	Workforce/Team (Includes; skills, training, qualifications, communication, and responsibilities)		
	Interface *(Involves; data handling, information exchange with secondary care, working with pharmacies and OOH providers)*		
	Patient Care/Involvement *(includes; patient education and participation)*		

### Consensus process

We used the RAND/UCLA Appropriateness Method [8,19], which involves combining a systematic summary of available scientific evidence with the collective judgement of experts. The Method is an established practice for the development of health indicators [8,20,21]. A consensus opinion is derived from the group, with individual opinions forming a refined, aggregated, group opinion.

We recruited nine internationally-recognised experts who had expertise in patient safety in general practice. A wide range of perspectives and personal characteristics were represented with participants coming from the US, England, Scotland, the Republic of Ireland, Switzerland, Germany, Belgium, and the Netherlands. The group contained seven men and two women with seven GPs, one pharmacist, and one academic (implementation science) on the panel. It was estimated that each member of the panel committed 3 days of work to the consensus-building exercise.

This study adhered to the RAND/UCLA Appropriateness Method [19] by conducting a two-round consensus process. In round one, which was conducted by email in June 2012, panel members were asked to consider each statement on its own merits using the summarised evidence for each as well as their own experience and knowledge. We asked panellists to separately rate the clarity, or lack of ambiguity, of each statement as well as the necessity of including it in the general practice patient safety toolkit that would be applicable to primary care settings in any country. Participants were also invited to provide alternative wordings for the statements and were asked about the appropriateness of different aspects of our operational definition of patient safety (as shown in the taxonomy in Table 3). In round two, panellists met for a 1-day face-to-face meeting in June 2012, under the chairmanship of two moderators (SC, AA), both with previous experience of chairing expert panels for safety indicators [14] and one with extensive experience of chairing expert panels for indicator development for the Quality Outcomes Framework [20]. Panellists discussed each statement in turn as a group and then re-rated them on individual rating sheets. These round-two rating sheets included the panellist’s own rating on round one, and for comparison, presented the frequency distribution for the ratings (anonymised) and the overall panel median rating from round 1. During round two, panellists also had the option to propose alternative wordings for statements, which they would then refine by consensus decision.

In both rounds, panellists were asked to rate each statement on a 9-point scale. In round 1, panellists rated ‘clarity’ and the ‘necessity to include the item in a general practice patient safety toolkit’. A clear item was defined as a ‘tool/indicator/criterion that was expressed in clear, precise and unambiguous language’. In round 2 the panellists re-rated ‘necessity’ and also rated ‘feasibility’. The necessity rating scale related to any general practice in any healthcare setting/country. The feasibility scale allowed participants to rate how feasible it would be to collect reliable data within UK settings, as this is the setting in which the toolkit will be initially applied.

### Data analysis

There are two aspects to the rating process for each scale for each scenario within a consensus technique: the overall panel median rating and also the level of agreement or consensus within a panel [8]. The level of consensus within the panel for each scale for each statement was calculated, adhering to the RAND/UCLA Appropriateness Method [19]. Agreement signified that 80% or more of panellists’ ratings were within the same 3-point region as the observed median. To be included in the final set of measures, statements had to achieve an overall panel median score of greater than or equal to 7 on the necessity scale, with no more than two panel members rating the indicator outside the 3-point distribution around the median. For example, if the median score was 7, no more than two panel members gave a score of <6 or >8. Results are presented for the final (round two) ratings only.

## Results

A total of 205 statements, summarising the tools, sets of indicators or individual indicators identified in the review, were rated in round 2. The full list of indicators can be obtained from the authors upon request. Summary statistics for these items are provided in Table 4, which shows the number and percentage of statements that were considered ‘necessary’ and the number percentage of items that were considered ‘feasible’ for each of the major categories (structures, processes, and outcomes). Table 4 shows that slightly more than half (56%) of the statements that covered structures were considered ‘necessary’. However, a little less than half of the statements in the Processes (48%) and Outcomes (43%) categories were considered ‘necessary’ by the panel. Most of the Items in the Structures and Processes categories were considered ‘feasible’ but the percentage of statements in the Outcomes category that were ‘feasible’ was only 29%. Generally, statements were rated equivocal or necessary with only a very small number rated unnecessary or unfeasible. As can be seen in Table 4, very few items were rated in the lowest tertile (1-3) on the necessity scales (n = 3 or 1.5%) and none on the feasibility scale (n = 0).

**Table 4:** Summary of necessity (and UK feasibility) ratings for round 2

	Necessity**	Feasibility***
Structures/Organisational*		
Indicators (n)	142	138
Necessary (%)	56	----
Feasible (%)	-----	54
Median 1-3 (n)	3	0
Median 4-6 (n)	48	49
Median 7-9 (n)	91	89
Agreement (%)	81	77
Equivocal (%)	18	22
Disagreement (%)	1	1
*Clinical Processes*		
Indicators (n)	48	48
Necessary (%)	48	----
Feasible (%)	----	65
Median 1-3 (n)	0	0
Median 4-6 (n)	24	17
Median 7-9 (n)	24	31
Agreement (%)	92	73
Equivocal (%)	8	27
Disagreement (%)	0	0
*Outcomes*		
Indicators (n)	14	14
Necessary (%)	43	----
Feasible (%)	----	29
Median 1-3 (n)	0	0
Median 4-6 (n)	7	7
Median 7-9 (n)	7	7
Agreement (%)	64	29
Equivocal (%)	21	71
Disagreement (%)	14	0

*Indicators were divided into three main categories: Structures/Organisational, Clinical Processes, and Outcomes

**One item was not rated by a sufficient number of panellists to obtain a median score

***Five items were not rated by a sufficient number of panellists to obtain a median score

The Additional file 2 shows that there was a total of 101 ‘necessary’ items (49% of the total statements), which were rated as necessary for inclusion in the toolkit, with the median necessity score for each item provided in brackets. Those statements that were rated with an overall panel median of 9 with agreement on the necessity scale are in bold. Where a re-worded statement during round 2 achieved a higher score on the necessity scale than the original statement, only the re-worded statement is included in the file labelled Additional file 2. The total number of feasible items for UK settings was 104 (51% of the total statements). Some statements were rated both necessary and feasible (n = 76, 37% of total) with 51 (36% of total) statements in the Structures category, 21 (44% of total) items in the Processes category, and 4 (29% of total) items in the Outcomes category considered both necessary and feasible. Statements that were considered necessary and feasible are shown in italics in the file labelled Additional file 2.

## Discussion

Understanding the epidemiology of safety in general practice needs addressing as rigorously as in hospitals [4]. There is a recognised lack of tools available to prevent, monitor and improve patient safety in primary care settings [2]. The epidemiology of patient safety in such settings is based largely on exploratory studies or estimates and focused predominately on prescribing. This study aimed to produce a set of patient safety tools and indicators for use in a ‘toolkit’ of patient safety measures for general practices. It provides the first attempt at identifying tools and sets of indicators that are necessary for inclusion in a general practice patient safety toolkit in any healthcare setting worldwide, covering issues related to structure, processes and outcomes. In addition, it provides ratings of the feasibility of collecting data using these tools in one country, the UK, a setting with highly sophisticated clinical computer systems and data coding in general practice. This study shows that there are a range of tools or instruments, derived mostly from the US and UK, that focus mainly on prescribing, trigger tools and safety culture in general practice. Good examples of such tools are the IHI Outpatient Adverse Event trigger tool [22] and the Safety Attitudes Questionnaire (ambulatory version) from the USA [23] and the RCGP prescribing indicators from the UK [14].

Most safety incidents can be categorised into four main areas: diagnosis, prescribing, communication between health care providers and patients, and organisational issues (i.e. safety climate/training, event reporting). Significantly, 20% of errors could have serious consequences [24]. Set within the context of the volume of healthcare interactions in general practice, this is a significant priority. The toolkit of safety measures identified in this study to date addresses all of these issues. Many are underpinned by the need for accurate and reliable health informatics [25] including electronic health records in general practice and across the primary-secondary interface, good coordination between primary and secondary care and effective multi-professional teams. The use of computerized provider order entry, medication reconciliation and clinicians working with clinical pharmacists to reduce adverse drug events have, for example, been emphasised as patient safety strategies that could be adopted in the US now [21]. The literature review conducted for this project, which will be published separately, revealed that many studies were not included in the AMA report [2]. Our final set of tools and indicators includes existing instruments on safety culture, trigger tools and prescribing. Patients themselves are underutilised in the safety processes of healthcare [26] so the toolkit also advocates the use of a Patient Reported Outcome or Experience Measure. It is imperative to involve patients actively as co-producers of safety and in the development of patient engagement and involvement strategies [27]. Obtaining informed consent to improve patients’ understanding of the potential risks of procedures is a strategy that should be adopted [21].

This set of tools and indicators has resonance with the findings of the recent review by the Health Foundation in the UK, which emphasised that there can be no one single measure of patient safety and the importance of knowing what methods, tools and indicators are currently being used in primary care to measure patient safety [7]. The literature review for the project identified 118 tools and hundreds of indicators. The file labelled Additional file 2 shows that the tools and indicators that were rated highly addressed mostly issues of structure and to a lesser extent processes. However, to paraphrase Donabedian [28], while good structure does not guarantee safe care it provides a greater opportunity to deliver it. Few tools or indicators of outcome were rated as necessary for inclusion but there was a relatively small pool of indicators about outcomes available from the literature. Although we must first be able to measure the correct things accurately, the ultimate value of such a toolkit is not to measure but to improve safety and prevent harm. This is especially important as there are virtually no credible studies on how to improve safety in primary care [4]. Moreover, there is an absence of guidance or recommendations of how available tools can be used in combination in routine clinical settings to help family practitioners, staff and patients to measure, and hence improve, patient safety.

We have presented all the statements felt necessary for inclusion in a general practice safety toolkit, as well as those felt to be feasible to collect data reliably in the UK, in order to make our toolkit as applicable as possible to the widest range of primary health care settings. While the ratings of feasibility relate to UK settings, many of the statements originated from US publications, which were felt not to be applicable to the UK (41% of the total published output of the systematic review). We have combined individual statements to produce a preliminary checklist consisting of items relating to information flow (both within the practice and between the practice and other providers), providing safety information about the practice, and achieving safer prescribing by working with patients. We produced the preliminary checklist in response to areas that we identified from our literature review as supported by relatively weak published evidence but in areas rated necessary by the panel, such as the handling of test results. We chose deliberately to exclude items related to well- established legal precepts, such as health and safety legislation and infection control, as practices should already be achieving the goals contained in these documents. The preliminary checklist attempts to bridge the gaps in our toolkit in relation to our taxonomy of patient safety.

This study adhered to a validated systematic consensus method [8]. Although the RAND/UCLA approach has been applied successfully for a variety of purposes, such as clinical appropriateness criteria [23], prescribing indicators [14] and quality indicators [20], we are not aware of any attempt to apply the method to identify a set of measures for patient safety in general practice. Ratings from such consensus techniques have high face validity; however, this is a minimum prerequisite for any measure and developmental work is needed to provide empirical evidence for acceptability, feasibility, reliability, sensitivity to change and validity [8,24,29]. The work presented in this paper forms phase one of the study. The tools and sets of indicators identified will be subjected next to prospective validation and empirical testing within samples of English general practice using observational designs.

## Conclusions

To improve patient safety we need to determine how to measure safety accurately, and identify ways of avoiding, preventing and ameliorating patient harm. This study, by focusing on tools and indicators of general practice patient safety, helps identify a range of measures that can be used by general practices to measure safety. The intended consequences of such an approach are to help quantify and measure existing safety levels and subsequently to develop interventions to improve safety outcomes in general practice. To meet this aim we must understand and meet the needs of service providers, patients and the public. The success of such a toolkit will be predicated on engaging with and helping practice staff deliver safety improvements, which are aligned to their own identified needs. Our intention is to develop and test tools that can lead to interventions akin to a list of top strategies for adoption in general practice, not unlike what has been done in hospital settings [21]. On-going evaluation will further our understanding of how best to implement this set of tools and indicators within general practices.

### What is already known on this subject?

There is a recognised lack of tools available to prevent, monitor and improve patient safety in primary care settings.

Understanding the epidemiology of safety in general practice needs addressing as rigorously as in hospitals.

### What this study adds?

We developed and tested tools that can lead to interventions to improve patient safety outcomes in general practice akin to what has been done in hospital settings.

The intended consequences of such an approach are to help quantify and measure existing safety levels and subsequently to develop interventions to improve safety outcomes in general practice.

## Competing interests

The authors declare that they have no competing interests.

## Authors’ contributions

Professors Campbell and Avery had full access to all of the data in the study and take responsibility for the integrity of the data and the accuracy of the data analysis. Study concept and design: BB, RS, AA and SC. Acquisition of data: BB, RS, AA and SC. Analysis and interpretation of data: BB, RS, AA and SC. Drafting of the manuscript: BB, RS, AA and SC. Statistical analysis: BB and RS. All authors read and approved the final manuscript.

## Authors’ information

The lead author affirms that the manuscript is an honest, accurate, and transparent account of the study being reported; that no important aspects of the study have been omitted; and that any discrepancies from the study as planned (and, if relevant, registered) have been explained.

## Pre-publication history

The pre-publication history for this paper can be accessed here:

http://www.biomedcentral.com/1471-2296/15/110/prepub

## Supplementary Material

Additional file 1 **Three stem search strategy.** The starting point for determining the search terms used in the review was a 3 point definition of our search question and exploration of Medical Subject Heading (MeSH) terms resulting in the following three stems. Click here for file

Additional file 2 **Statements rated necessary for inclusion in a general practice patient safety toolkit relevant to any country.** *Statements with median of 9 in bold. (=13 in total) Statements considered both necessary and feasible are in italics. () number in brackets = median rating by the panel [30,31,32,33,34]. Click here for file
