# Supplementary material for: Tools for measuring patient safety in primary care settings using the RAND/UCLA appropriateness method
Source: BMC Fam Pract. 2014 Jun 5;15:110. doi: 10.1186/1471-2296-15-110 (PMC4060097; doi:10.1186/1471-2296-15-110)
Supplement: Additional file 1 — Three stem search strategy. The starting point for determining the search terms used in the review was a 3 point definition of our search question and exploration of Medical Subject Heading (MeSH) terms resulting in the following three stems. [file 1471-2296-15-110-S1.docx]

**Appendix 1 Three stem search strategy**

The starting point for determining the search terms used in the review was a 3 point definition of our search question and exploration of Medical Subject Heading (MeSH) terms resulting in the following three stems.

Setting

"Family physician" OR "primary care" OR "family practice" OR "general practice" OR "ambulatory care" OR "ambulatory health" OR "ambulatory health-care" OR "ambulatory healthcare" OR "community health" OR "community healthcare" OR "community health-care" OR "primary health" OR "primary healthcare" OR "primary physician" OR "primary health-care" OR generalist OR "family medicine"

Safety synonyms

"administration error" OR "administration errors" OR "dispensing error" OR "dispensing errors" OR "medication error" OR "medication errors" OR "medical mistake" OR "medical mistakes" OR "prescription error" OR "prescription errors" OR "prescribing error" OR "prescribing errors" OR "prescribing fault" OR "prescribing faults" OR "medical error" OR "medical errors" OR malpractice OR "safety" OR "safety-culture" OR "adverse event" OR "adverse events" OR "adverse effect" OR "adverse effects" OR "adverse reaction" OR "adverse reactions" OR harm OR harms

Types of tools

scale OR scales OR survey OR surveys OR questionnaire OR questionnaires OR instrument OR instruments OR indicator OR indicators OR "outcome assessment" OR "outcome assessments" OR "patient reported outcome" OR "patient reported outcomes" OR "patient experience" OR "patient experiences" OR "practice guideline" OR "practice guidelines" OR "quality assurance" OR tool OR tools OR toolkit OR toolkits
