# Supplementary material for: Tools for measuring patient safety in primary care settings using the RAND/UCLA appropriateness method
Source: BMC Fam Pract. 2014 Jun 5;15:110. doi: 10.1186/1471-2296-15-110 (PMC4060097; doi:10.1186/1471-2296-15-110)
Supplement: Additional file 2 — Statements rated necessary for inclusion in a general practice patient safety toolkit relevant to any country. *Statements with median of 9 in bold. (=13 in total) Statements considered both necessary and feasible are in italics. () number in brackets = median rating by the panel [30,31,32,33,34]. [file 1471-2296-15-110-S2.docx]

**Appendix 2 Statements rated necessary for inclusion in a general practice patient safety toolkit relevant to any country**

*Statements with median of 9 in bold. (=13 in total)

Statements considered both necessary and feasible are in italics.

() number in brackets = median rating by the panel

*Structures*

**ORGANISATIONAL - BACKGROUND SYSTEMS – DATA HANDLING including:
1) information flow into and out of the practice
2) follow-up to tests/investigations
3) clinical handover**

1. ***Where an incoming result, report or investigation requires follow-up there are systems in place to ensure it occurs. [9]****
2. ***The practice has procedures that ensure incoming clinical information is seen by a GP in the practice to view and action before or after being filed in the patient’s record [9]***
3. The percentage of patients discharged from hospital, on long term medication, with a record of a medication review by the practice within 72 hours of the date of discharge (not necessarily face to face) [8]
4. The toolkit should include the general idea of toolkits such as the LIMM^30^ [8]
5. The percentage of patients discharged from hospital that have a follow up appointment with their GP [7]
6. *The percentage of patients admitted to hospital for whom there is a record of all medication and clinical summary information available to the admitting hospital within 24 hours. [8]*
7. *The practice has and operates a policy regarding the management of patient care following discharge from hospital [7]*
8. *The practice has a system on how to identify and record follow up patients who have a diarised activity for patient safety critical issues recorded in their notes including for example follow up of blood tests such as PSA, INR etc. [8]*
9. The practice has a process or system for informing patients, or where appropriate, families and carers, of abnormal investigation results [8]
10. *The practice can produce a log of histology specimens sent in the previous 12 months [8]*
11. ***The practice has a system and protocol in place for tracking results [9]***
12. *The practice has a protocol and can demonstrate a robust system for managing lab results [8]*

**ORGANISATIONAL - BACKGROUND SYSTEMS – Incident reporting / error classification system / adverse events**

1. *The practice monitors errors and adverse events using a trigger tool on a routine basis [8]*
2. *The Patient Safety Toolkit should include a system for reporting adverse drugs reactions to regulatory authorities. [8]*
3. The practice monitors errors and adverse events using a trigger tool on a routine basis for adverse events related to non-cancer opiate prescribing [7]

**ORGANISATIONAL - BACKGROUND SYSTEMS – Incident reporting / error classification system / adverse events**

1. *The practice has a patient safety reporting system in place and used routinely as part of day-to-day practice [7]*

**ORGANISATIONAL – PRACTICE MANAGEMENT – policies including governance, hazard protocols, infection control, monitoring staff, information for patients.**

1. *The practice has clinical protocols for infection control [8]*
2. *The practice has evidence to demonstrate the actions it takes to prevent and control infection and that it meets the requirements of National Specifications for cleanliness as applicable to primary care [8]*
3. *The arrangements for instrument sterilisation and decontamination or disposal of disposable instruments comply with national guidelines as applicable to primary care [8]*
4. *Information on the practice policies and procedures, and local facilities and services is provided to guide locums and other temporary clinical staff who work in the premises [8]*
5. *There is an office procedure manual covering the administrative procedures and systems for the daily running of the practice to which team members have access that are discussed and agreed by team members and are reviewed at least annually [8]*
6. ***There is a system for ordering, storing, handling and recorded dispensing of controlled drugs, which is reviewed and inspected on at least an annual basis [9]***
7. *Non-collection of prescriptions held by the practice are monitored and followed-up by the practice. / Medications which are not claimed by patients are a trigger for review [7]*
8. *The Patient Safety Toolkit should include a general practice survey on patient safety [8]*
9. *The Patient Safety Toolkit should include age specific prescribing alerts for elderly patients on alert burden and frequency of prescribing contraindicated medications [8]*
10. *The Patient Safety Toolkit should include a temperature log is maintained for refrigerators [7]*
11. *The Patient Safety Toolkit should include a protocol for the storage and disposal of hazardous waste materials [7]*
12. *The Patient Safety Toolkit should include a protocol which is implemented for the storage and usage of vaccines [8]*

**ORGANISATIONAL - ELECTRONIC HEALTH RECORD / INFORMATICS / - use of information systems Computerized physician order entry**

1. *This area/issue (electronic health records/informatics) should be included in a general practice Patient Safety Toolkit (a good general example of an IT measure in patient safety is the SEMI-P)^31^ [8]*
2. *The practice uses an electronic health record [8]*
3. *The practice uses a computer-based clinical decision support system (CDSS) as part of routine practice [8]*
4. *The practice uses a computerised ‘query set’ / Computerized physician order entry (CPOE) alerts for interrogating general practice computer systems to identify important hazard, allergy, drug interaction and contradiction alerts, which cannot easily be overridden [8]*
5. The percentage of overrides of prescribing critical alerts for which there is an audit trail showing the reason for the override [7]
6. *The practice uses an electronic- or e-prescribing system of prescribing for all prescriptions Computerized physician order entry (CPOE) system for medications (electronic- or e-prescribing system) [8]*
7. The percentage of all prescriptions generated in the last 12 months using an electronic/computerised or e-prescribing system of prescribing [7]
8. *The practice participates in the review of Epact (Electronic Prescribing Analysis and Cost )or similar data, to review clinical appropriateness of prescriptions [7]*
9. *The practice uses an up-to-date electronic drug interaction database / tool routinely on all computers used for prescribing [8]*
10. *The practice uses an electronic vaccine adverse event surveillance and reporting system (Vaccine Adverse Event Reporting System (VAERS)^32^ [7]*
11. The practice has up to date clinical summaries in at least 80% of patient records [7]
12. *The Patient Safety Toolkit should include a computerized alert for prescribing to patients with renal failure [8]*
13. ***The practice can produce accurate and up to date medications lists for 100% of its patients*** ***[9]***

**ORGANISATIONAL - WORKFORCE – including skills/training, communication, lines of responsibility, occupational health**

1. Responsibilities for management and administration, and accountability and reporting structures, within the team are clearly defined and understood by team members [8]
2. All members of the team are suitably trained, supervised and keep their skills up to date, and only carry out consultations, treatments and procedures which are within their competence [8]
3. *All team members have training at induction and are refreshed as appropriate on the principles of the Data Protection Act [8]*
4. *All staff including GPs are trained to make safe use of their clinical systems [7]*
5. *There is a record of all practice-employed clinical staff having attended training/updating in basic life support skills in the preceding 18 months [7]*

**ORGANISATIONAL - LEARNING ORGANISATION – needs of population**

1. *The practice has a surveillance system for 'at-risk' children when known to the practice in place to ensure that when a child has been recognised as being at risk this can be easily identified from their record. [7]*

**ORGANISATIONAL - LEARNING ORGANISATION - SAFETY CULTURE / CLIMATE**

1. *The practice has administered a safety climate questionnaire to all staff in the last 3 years [8]*
2. *The Patient Safety Toolkit should include the Manchester Patient Safety questionnaire (MAPSAF)^33^ [7]*

**ORGANISATIONAL - LEARNING ORGANISATION – complaints related to patient safety incidents**

1. ***The practice has an operational complaints protocol which ensures compliance with new General Medical Services Contract, acts appropriately in response to concerns or complaints, where appropriate ensuring improvements in care and ensuring that patients, relatives or carers who complain are not discriminated against [9]***
2. ***The practice ensures that information about how to make a complaint is readily available to people who use services. People who make a complaint are given information promptly about its investigation, any changes that will result, and are given an apology if appropriate. [9]***
3. *The practice conducts an annual review of patient complaints and suggestions to ascertain general learning points which are shared with the team [7]*

**ORGANISATIONAL - LEARNING ORGANISATION – Significant Event Audits**

1. **The practice builds a safety culture and takes action to implement learning by operating a policy to identify and learn from all patient safety incidents, significant events and incidents and recommendations and alerts from external bodies to share learning points with all team members and also any relevant outside agencies [9]**
2. *The Patient Safety Toolkit should include generic Significant Event Analysis tools [8]*
3. *The practice reflects on case studies of error and evidence of implementation of change in practice on a regular basis [8]*
4. ***The practice has undertaken a minimum of 12 significant events about patient safety in the preceding 3 years [9]***
5. *The practice has undertaken a minimum of 3 significant event reviews within the preceding year [8]*

**ORGANISATIONAL - LEARNING ORGANISATION -
safety improvement; including adherence to protocols, guidelines, training, attitudes to patient safety**

1. *The practice has a policy in place that supports health care staff after an error disclosure or adverse event [8]*
2. *The team regularly audits its work on patient safety, demonstrating the full audit cycle and the application of criteria and standards where appropriate, and uses this information to evaluate and set goals about how their service could be improved [8]*
3. *Team meetings take place quarterly to discuss clinical and safety issues (with clinical and safety issues always separate items on the agenda) and policies where all provider and attached team members are invited to attend and contribute [8]*
4. The practice investigates performance concerns and risk management [7]
5. Each practicing GP at the practice reflects on their ‘most serious errors in treatment’ and about the consequences for themselves in the previous 12 months [8]
6. *The practice uses rational prescribing outcomes and indicators for GP's CME (Continuing Medical Education) programmes [7]*
7. *The practice works with pharmacists in interactive educational meetings to discuss elderly patients exposed to polypharmacy [7]*
8. The Patient Safety Toolkit should include the practice undertaking root cause analysis with patients of adverse drug events to develop patient-centered medication safety strategies [7]

**ORGANISATIONAL - INTERFACE**

1. The practice works with pharmacies using a random selection of patients to identify potential Adverse Drug Reations [7]
2. The practice works with pharmacies to review (and audit) all prescribing of controlled drugs [7]
3. The practice engages in collaborative medication reviews with local pharmacists [8]

**ORGANISATIONAL - PATIENT/CARER ROLE & INVOLVEMENT - Including patient participation**

1. The practice encourages people to be involved in the how the service is run. The practice has effective methods of working with, involving and communicating with patients and carers to plan, develop and implement services. It also encourages suggestions and feedback from individual patients with this feedback responded to by the practice [7]
2. The percentage of patients on >4 repeat medications with a record of being asked to bring their medications or a list of their medications to their next recorded appointment [7]
3. The practice works with patients to ensure medication list accuracy (medication reconciliation) upon hospital referral / admission [8]
4. The practice works with hospital staff to ensure medication list accuracy (medication reconciliation) upon hospital admission and hospital discharge [8]
5. The practice uses clinical systems that patients can use to check accuracy of their medication record and promote compliance [7]

*Processes*

**CLINICAL PROCESSES - DIAGNOSIS**

1. *The Patient Safety Toolkit should include a diagnosis incident reporting tool [7]*

**CLINICAL PROCESSES - TREATMENT - PRESCRIBING – Tools/ set of prescribing indicators**

1. ***The practice uses a PIP (potentially inappropriate prescribing) tool to assess the safety of prescribing [9]***
2. *The percentage of patients prescribed >5 items with a record of a medication review within the last 6 months [7]*
3. *The percentage of patients prescribed >5 items with a record of a medication review within the last 12 months [8]*
4. *The Patient Safety Toolkit should include the RCGP (Royal College of General Practice) prescribing indicators^14^ [7]*

**CLINICAL PROCESSES - TREATMENT - PRESCRIBING – preventing harm**

1. *The practice uses treatment indication in an integrated electronic prescribing systems [8]*
2. *A medication review is recorded in the records in the preceding 15 months for all patients being prescribed repeat medicines. [8]*
3. *A medication review by a clinical pharmacist is recorded in the records in the preceding 15 months for all patients being prescribed 7 or more repeat medicines [7]*
4. *The percentage of patients who have had a medication review in the last 15 months if they are recorded as having five or more (repeat) medications [8]*
5. The percentage of patients who have had a medication review in the last 15 months if they are recorded as having ≥12 doses per day [7]
6. *The percentage of patients who have had a medication review in the last 15 months if they are recorded as having three or more chronic diseases [7]*
7. ***The percentage of patients who have had a medication review in the last 15 months if they are recorded as having the presence of a drug requiring therapeutic drug monitoring (TDM). [9]***
8. *All patients prescribed ≥4 repeat medications have a record of a mediation reconciliation review in the preceding 15 months [7]*
9. *A review of a sample of patients at 'high risk' of medication safety problems is conducted [8]*
10. *The Patient Safety Toolkit should include a system by which the practice works with all local nursing homes to maintain up-to-date drug register for all residents, which is available to primary care staff [7]*
11. *The percentage of patients residing in a care/nursing home registered with the practice that have had a medication review in the previous 6 months [8]*
12. ***The practice has routine operation computerized physician order entry (CPOE) prescribing alerts for the following; allergies, duplicates, (age related factors), (disease related factors) [9]***
13. *The Patient Safety Toolkit should include the PINCER Trial indicators^34^ [7]*
14. *The Patient Safety Toolkit should include high risk prescribing alerts (defined as potentially inappropriate prescribing of drugs to primary care patients who are particularly vulnerable to adverse drug events). [8]*

**CLINICAL PROCESSES - TREATMENT - PRESCRIBING – Patient role**

1. *The patient safety toolkit contains a prescribing PROM (Patient Reported Outcome Measure) [7]*
2. *The practice works with patients to ensure medication list accuracy (medication*

*reconciliation) upon hospital referral /admission [8]*

**CLINICAL PROCESSES - TREATMENT - minor surgery**

1. *The practice keeps a record or log of minor operations which will have the following information recorded; 1) date; 2) patient name; 3) procedure performed; 4) team members involved; 5) whether a specimen was sent for histology; 6) patient consent; 7) complications; 8) patient informed of result [7]*

*Outcomes*

**OUTCOMES – PATIENT REPORTED OUTCOME/EXPERIENCE MEASURES**

96) *The toolkit should contain a PROM [8]*

97) The Patient Safety Toolkit should include patients’ perceptions of the way in which physicians/practices have handled an error/incident/adverse event that the patient had been exposed to [7]

**OUTCOMES – MORTALITY**

98) **All unexpected deaths should have an SEA recorded [9]**

**OUTCOMES – OUTCOMES- Significant Event Audit (SEA)**

99) *The percentage of Significant Event Audit reports where the care provided was judged to be unsatisfactory by a trained reviewer [7]*

100) *The percentage of Significant Event Audit reports where the report was judged to be unsatisfactory by a trained reviewer [8]*

**OUTCOMES – COMPLAINTS**

101) *The practice conducts an annual review of patient complaints and suggestions to ascertain general learning points which are shared with the team [7]*
